# MicroRNAs as Biomarkers in Cancer

**DOI:** 10.3390/diagnostics3010084

**Published:** 2013-01-16

**Authors:** Kamini Sundarbose, Reena V. Kartha, Subbaya Subramanian

**Affiliations:** 1 Division of Basic and Translational Research, Department of Surgery, University of Minnesota, Minneapolis, MN 55455, USA; E-Mail: kaminisanjev@gmail.com; 2 Department of Experimental and Clinical Pharmacology, University of Minnesota, Minneapolis, MN 55455, USA; E-Mail: rvkartha@umn.edu; 3 Masonic Cancer Center, University of Minnesota, Minneapolis, MN 55455, USA

**Keywords:** microRNA, serum biomarkers, cancer, diagnostic and prognostic markers

## Abstract

MicroRNAs (miRNAs) are small non-coding RNA molecules, which in recent years have emerged to have enormous potential as biomarkers. Recently, there have been significant developments in understanding miRNA biogenesis, their regulatory mechanisms and role in disease process, and their potential as effective therapies. The identification of miRNAs as biomarkers provides possibilities for development of less or non-invasive and more specific methods for monitoring tumor growth and progression. This review summarizes the recent developments in methods to detect and quantitate miRNAs in body fluids and their applications as biomarkers in cancers. The prospect of miRNAs as potential diagnostic and prognostic biomarkers with clinical applications is significant as more evidence points to their central role in cancer pathobiology.

## 1. Introduction

MicroRNAs (miRNAs) are a class of non-coding small RNAs (18–24 nucleotides), which are endogenously stable and evolutionarily conserved molecules. The major role of miRNAs appears to be in acting as crucial switches in regulating posttranscriptional gene expression. In recent years, miRNAs have been identified to play an important role in many physiological and pathological conditions [1,2,3,4]. Particularly, miRNA deregulation in various cancer types has been extensively studied. For example, deregulations in miRNAs expression have been identified to play a role not only in major cancers like lung [5], breast [6] and prostate [7] but also in rare cancers like waldenstrom macroglobulinemia [8] and cholangiocarcinoma [9]. In this review, we summarize the research advancements of using miRNAs as circulating biomarkers in cancers. The prospect of miRNAs as potential diagnostic and prognostic biomarkers in a clinical setting is significant as more evidence points towards a central role of miRNAs in cancer development and progression.

## 2. MicroRNAs and Cancer

microRNAs play a fundamental but significant role in cellular functions [10,11,12,13,14,15]. miRNA biogenesis and the mechanisms underlying miRNA mediated gene regulation are described elsewhere [16,17,18]. Since the discovery of miRNAs in *C. elegans*, about 1,500 miRNAs have been reported in humans [19]. Earlier studies established the function of miRNAs such as lin-4 and let-7 during embryogenesis and developmental control in *C. elegans* when complex gene regulations occur. Biochemical characterizations showed that these small RNAs block protein synthesis and/or regulate mRNA stability [20,21]. miRNAs have been found to regulate the expression of about 50% of the human coding gene transcripts [20]. The gene regulatory role of miRNAs suggests their important role in physiological functions [21] and pathological conditions [2,3]. Furthermore, single nucleotide polymorphisms in the 3’UTR of mRNAs may affect miRNA-mediated regulation and have been associated with certain cancer risk [22].

Pioneering studies by Calin *et al. *showed the biological relevance of miRNAs in cancer development; miR-15 and miR-16 were significantly downregulated in chronic lymphoid leukemia (CLL) through deletions in chromosome 13q14 locus. These earlier observations also revealed 52% of miRNA genes to be located in genomic regions, which are frequently altered in cancer. These results were further validated by mapping which revealed 186 miRNAs located at fragile sites and genomic regions [23]. Since this discovery, miRNAs have been implicated in many different types of cancers including the influence of miRNAs in neoplastic transformation, progression and patient outcomes [24,25,26,27,28,29]. miRNAs act as oncogenes or tumor suppressors taking the lead role in regulating cancer pathways. The target genes regulated by the miRNAs determine their role as oncogenes or tumor suppressors.

### 2.1. MicroRNAs as Oncogenes

The oncogenic potential of miRNAs has been well documented. An earlier study showed that B-cell integration cluster (BIC) accelerates *MYC* mediated growth of lymphomas [30]. Subsequent characterization of BIC gene revealed this region to harbor the primary transcript for miR-155. In pancreatic cancer, miR-155 is expressed at high levels and targets tumor suppressor *TP53INP1*, which is a stress induced-gene thus impairing stress response and resulting in decreased apoptosis [31]. Similarly, miR-17-92 cluster was identified as a potential oncogene since its expression along with cMYC accelerated the development of B-cell lymphoma in mouse models [32]. Likewise, miR-21 is significantly upregulated in many cancers and its validated targets includes tumor suppressor genes such as *PDCD4*, *PTEN* and *TIMP3*. miR-21 overexpression thereby inhibits apoptosis and promotes cell survival. Oncogenic miRNAs such as miR-221 and miR-222 inhibit *CDKN1B* and *CDKN1C *genes [25], which are important cell cycle regulators*. *Similarly*, *miR-182 targets FOXO1 and FOXO3 transcription factors and protects from oxidative stress and cell death [26,27]. 

### 2.2. MicroRNAs as Tumor Suppressors

miRNAs also function as tumor suppressors in different cancer types by regulating the oncogenic process and preventing tumor development. Kefas *et al.* documented the association of EGFR and AKT pathway activation with aggressiveness of glioblastoma [28]. The suppression of the AKT pathway and inhibition of EGFR by miR-7 resulted in decreased proliferation, survival and invasiveness providing evidence for the tumor suppressor effect of this miRNA. Similarly, in a study comparing primary melanoma and benign nevi, the authors showed the downregulation of let-7 family miRNAs in primary melanomas suggesting their role as tumor suppressors. Exogenous expression of let-7b in primary melanoma cells decreased the anchorage independent growth and inhibited cell cycle progression [29]. The increased expression of let-7b also suppressed *CDK4* and cyclins (*D1, D3 & A*), which are implicated in the development of melanoma. In a recent study by Dong *et al*. [33] on uveal melanoma, which is the most common primary form of the intra ocular tumor in adults, the transfection of uveal melanoma cells by miR-34b/c caused cell cycle arrest resulting in significant reduction of melanoma cell growth and migration. The miR-34b/c has been identified to target the *MET* proto-oncogene in uveal melanoma cells. Interestingly, many studies have demonstrated miRNAs as oncogenes or tumor suppressors by involving multiple molecular mechanisms and different signaling pathways. There can be a single target or multiple functionally relevant targets for a miRNA. For example, miR-155 is upregulated in Hodgkin’s lymphoma and targets genes such as *ZIC3*, *AGTR1*, *ZNF537*, *FGF7 *and *IKBKE* [34], while miR-206 is downregulated in rhabdomyosarcoma and targets *MET *onco-protein [35]. The understanding that miRNAs can function as oncogenes and/or tumor suppressors in cancers allows us to explore them as therapeutic targets for clinical application. Extensive studies are underway to establish them as biomarkers for cancer detection and progression.

## 3. MicroRNAs as Potential Biomarkers in Cancer

With our increasing knowledge on gene regulatory pathways, we have achieved certain milestones in our understanding of cancer signaling. However, there is an immense need for fail proof biomarkers that can detect cancer in its earlier stages and/or function as prognostic indicators. Currently, most cancer diagnosis depends on imaging techniques like computerized tomogram (CT), magnetic resonance imaging (MRI), ultrasonography, positron emission tomography (PET), mammogram and invasive studies like colonoscopy, bronchoscopy and biopsy. Even though imaging and invasive studies are extensively relied on in order to generate valid information regarding the cancer, there is a high possibility of false positive or false negative results. The imaging methods are not cost effective and invasive studies cause unnecessary discomfort for the patients. 

Further, the available biomarkers such as CA 125 for ovarian cancer, CA 19-9 for pancreatic cancer and CEA (carcino embryonic antigen) for colon cancer have poor sensitivity and specificity. Only 50% of patients with early stage ovarian cancer have elevated levels of CA125. In 2010, the National Institutes of Health advisory panel did not reissue the old protocol which advised women to begin yearly breast cancer screening once they attained the age of forty. Furthermore, the U.S. Preventive Services Task Force (USPSTF) recommendation on prostate cancer screenings (PSA) made available in October 2011 recommends discontinuing routine PSA tests due to poor sensitivity and specificity in distinguishing prostate cancer patients requiring surgery. Thus, there is a critical need for reliable biomarkers that allow precise monitoring of changes at the cellular level and the patient’s response to therapy. miRNAs have tremendous potential to be explored as diagnostic and prognostic biomarkers. miRNA expression is dynamic; many miRNAs are deregulated in early stages of tumor development and are upregulated during cancer progression exhibiting their potential for diagnostic utility [36,37]. The possibility of using combination of markers such as CA-19-9 and miRNAs significantly increase the diagnostic potential [38].

Resnick *et al.* compared levels of miRNAs (miR-21, -92 and -93) and the currently available biomarker for ovarian cancer, CA-125, and showed alteration in the level of these miRNAs in patients with ovarian cancer having normal levels of CA-125 [39]. With increasing implications of miRNAs in cancer development and progression, significant efforts are underway to use miRNAs as novel biomarkers with clinical applications [40] and as novel therapeutic targets for anti cancer drug resistance [41]. Another potential is in the diagnosis of pancreatic cancer, which still remains a cancer with poor prognosis with a median survival of 6 months to 1 year. Pancreatic cancer is asymptomatic in the early stages when their precursor lesions such as PanIN (pancreatic intraepithelial neoplasia) remain undetected. However, if these precursor lesions are detected, the potential for cure may be high. Several miRNAs have been identified in PanIN stages [42,43] of pancreatic cancer that allow us to investigate them as potential biomarkers.

## 4. Circulating MicroRNAs

Blood sampling from patients remain the least invasive method for identifying biomarkers. The presence of circulating miRNAs has been demonstrated in various disease conditions and the profiles vary with the degree of disease progression [44,45,46,47,48]. The presence of a specific set of circulating miRNAs in cancer condition is justified by passive diffusion due to high turnover of cells in cancer. Yu *et al.* showed differences between the metabolite profiles of plasma and serum. Concentrations of metabolite were generally higher in serum and provided higher sensitivity than plasma [49]. Initial studies to identify serum miRNAs in cancer were demonstrated by Lawrie *et al.*, in which they observed that miR-155, miR-210, and miR-21 were upregulated in diffuse large B cell lymphoma when compared to healthy controls [50]. Further, miRNA expression profiles have been generated using serum/plasma from patients diagnosed with common cancers such as acute leukemia [51], breast cancer [52], colorectal cancer [53], gastric cancer [54], glioblastoma [55], hepatocellular cancer [56], lung cancer [57], oral and squamous cancer [58], ovarian [39] and prostate cancer [40]. Recently, miRNAs have also been detected in numerous other body fluids (see below). 

With the increasing data about serum miRNAs as well the growing need for a good biomarker, which must also be easily accessible, the miRNAs in serum can be considered valid biomarkers for early detection, diagnosis and prognosis of various cancers [59,60,61,62,63,64]. As more interest has been drawn toward identifying circulating miRNAs in cancers, the biggest challenge currently encountered during the analysis of miRNAs in serum/plasma and other body fluids is the need for an internal normalization. Housekeeping genes like U6 or *GAPDH,* commonly used as internal normalization during miRNA analysis in cells and tissues, are not feasible in serum analysis since these controls are easily degraded and not detected in serum. Chen *et al.* [57] demonstrated a series of miRNAs existing in normal human serum and compared their levels to widely used U6, which was not stable in serum. They also showed the presence of miRNAs in the serum of rats, mice, calves, bovine fetuses and horse and the potential for using these miRNAs for normalization and comparative studies. In support of these observations, Mitchell *et al.* demonstrated the stability of miRNAs (miR-15b, miR-16, miR-19b and miR-24) occurring in normal human sera and their resistance to RNase digestion. The levels of these miRNAs are not altered during tumor growth and can possibly be considered as normalization controls [40]. Of these normally present miRNAs, miR-16 was widely present and was used as internal controls in serum miRNA studies [57]. However a recent study demonstrated high levels of miR-16 in red blood cells; thus it is possible that the presence of miR-16 in serum may in part be due to hemolysis of red blood cells [65]. This also raises the question of using miR-16 for normalization. As greater numbers of miRNAs are being detected in normal blood cells, to avoid the errors with internal control there is an essential need for a standard, normalization protocol when analyzing miRNAs from serum samples. This challenge is, for now, met by using spike-in synthetic *C. elegans* miRNA for internal normalization when using serum samples for miRNA analysis. Taken together, circulating miRNAs detected using validated methods can be valuable biomarkers for cancer diagnosis and prognosis. A comprehensive list of miRNAs identified as potential biomarkers in various cancer types are given in Table 1.

**Table 1:** miRNAs in various cancer types, their source and validated targets.

Cancer	Sample type	Comparison criteria	Methods	miRNAs	Target/mechanisms	Ref.
**Epithelial cancers**						
**Breast**	Tissue cancer cells	ER+, ER− tissues	Microarray, qPCR	miR-34b (↓)	miR-34b target cyclin D1 and JAG-1	[66]
	Whole blood	Tumor *vs*. normal	qPCR	miR-195 (↑) let-7a (↑) miR-155 (↑)	let-7a target *KRAS* and miR-155 target RhoA transforming growth factor and induces EMT	[67]
	Cancer cells	Cancer cell growth	Microarray	miR-21 (↑)	PDCD4	[68]
**Colorectal **	Tissue & serum	Tumor *vs*. normal	qPCR microarray	miR-17-3p (↑) miR-92 (↑)	miR-92 is elevated in plasma and can be used as a non-invasive molecular marker for screening	[53]
	Plasma	Tumor *vs*. normal	qPCR	miR-29a (↑) miR-92a (↑)	Promote cell proliferation, suppressed apoptosis, induce tumor angiogenesis and accelerated tumor progression	[69]
**Bladder**	Tissue & cancer cell lines	Tumor *vs*. normal	qPCR	miR-145 (↓) miR-30a-3p (↓) miR-133a (↓) miR-133b (↓) miR-195 (↓) miR-125b (↓) miR-199a (↓)	These miRNAs are downregulated and play the role of tumor suppressors by targeting KRT7, a common target with oncogenic function	[70]
**Glioblastoma**	Tissue	Tumor *vs*. normal, Different grades of malignancy	qPCR Northern blotting	miR-21 (↑) miR-221 (↑) miR-128 (↓) miR-181b (↓)	Knockdown of miR-21 triggered the activation of caspases leading to apoptosis. miR-221 & miR-222 repress expression p27Kip1. miR-181b triggered growth inhibition, apoptosis, and inhibited invasion	[71]
**Gastric**	Tissue & cell lines	Tumor *vs*. normal	qPCR	miR-409 (↓)	miR-409 target RDX and suppresses cell invasion and metastasis	[72]
**Lung**	Serum	Overall survival in NSCLC	sequencingqPCR	miR-486 (↑) miR-30d (↑) miR-1 (↓) miR-499 (↓)	miR-1 downregulates MET oncogene. Facilitates activation of Caspase 3, Caspase 7, and PARP-1 as well as depletion of MCL-1	[73]
	Plasma	Tumor *vs*. normal	qPCR	let-7f (↓) miR-20b (↓) miR-30e-3p (↓)	let-7 targets cMyc. miR-30e regulates of Ubc9	[74]
**Oral & squamous cell**	Tissue	Tumor *vs*. normal	qPCR	miR-184 (↑)	miR-184 alters cMyc expression and affects anti-apoptosis and proliferation of tongue SCC cells	[58]
	Tissue & saliva	Tumor *vs*. normal	qPCR	miR-31 (↑)	-	[75]
**Ovarian**	Serum	Tumor *vs*. normal	qPCR	miR-21 (↑) miR-29a (↑) miR-92 (↑) miR-93 (↑) miR-99b (↓) miR-126 (↓) miR-127 (↓)) miR-155 (↓))	miR-21 regulates PDCD4 and maspin miR-92 and miR-93 regulates TGFβ miR-29a potentially targets PTEN miR-127 regulates BCL6	[39]
**Prostate**	Plasma, serum, murine	Tumor *vs*. normal	qPCR	miR-141 (↑) miR-375 (↑) miR-107 (↑) miR-574-3p (↑)	induces abnormal cell division and proliferation and the development of aggressive prostate cancer	[76]
	Tissue & Serum	Metastatic, localized tumors vs. normal	qPCR	miR-141 (↑) miR-375 (↑)	regulates genes controlling cellular growth and proliferation	[77]
**Pancreatic**	Tissue	Tumor *vs*. normal	qPCR	miR-155 (↑) miR-203 (↑) miR-210 (↑) miR-222 (↑)	-	[78]
	Plasma	Tumor *vs*. normal	qPCR	miR-21 (↑) miR-155 (↑) miR-196a (↑) miR-210 (↑)	miR-21 targets PTEN and PDCD4 miR-210 affect DNA repair and genomic instability miR-155 target TP53INP1	[79]
**Hepatocellular**	Tissue & cell cultures	Tumor *vs*. normal	qPCR	miR-519d (↑)	miR-519d has inhibitory effect on CDKN1A/p21, PTEN and TIMP2 expression	[80]
**Endometrial**	Tissue	Tumor *vs*. hyperplasia *vs. *normal	qPCR	miR-200 family (↑)	Negatively regulates ZEB1 and ZEB2 and implicated in EMT	[81]
**Renal cell**	Tissue	Tumor *vs*. normal	qPCR Microarray	miR-122 (↑) miR-155 (↑) miR-210 (↑) miR-200c (↓) miR-335 (↓) miR-218 (↓)	-	[82]
**Melanoma**	Tissue & cell lines	Normal *vs*. cancer cell lines	Microarray	miR-193a (↓) miR-338 (↓) miR-565 (↓) miR-191 (↓) miR-193b (↑)	miR-193 is regulated by HNF-1a and p53; predicted targets for miR-191 include FZD5 and BDNF	[83]
**Thyroid **	Tissue	Tumor *vs*. Normal	qPCR	miR-187 (↑) miR-221 (↑) miR-222 (↑) miR-146b (↑) miR-155 (↑) miR-224 (↑) miR-197 (↑)	The oncogenic mutations in PCs, RET/PTC, BRAF, and RAS are all capable of activation of the MAPK pathway	[84]
**SARCOMAS**						
**Osteosarcoma**	Tissue & cancer cell lines	Tumor *vs*. normal	qPCR	miR-135b (↑) miR-150 (↑) miR-542-5p (↑) miR-652 (↑)	Pro-apoptotic EGR2 and P2X7 are targets of miR-150	[85]
	Tissue	Tumor *vs*. normal	qPCR Microarray	miR-17-92 (↓)	14q32 miRNAs (miR-544, miR-369-3p, miR-134 and miR-382) act cooperatively to destabilize cMYC and in turn, control expression of miR-17-92 miRNAs	[86]
**Leiomyosarcoma**	Tissue	Tumor *vs*. normal	qPCR Microarray CGH	miR-21 (↑) let7 (↑) miR- 27a (↑) miR-30a (↑) miR-23b (↑) miR-29b (↓) miR-32 (↓) miR-144 (↓) miR-212 (↓) miR-197 (↓)	Targets MAPK pathway genes	[87]
**Rhabdomyo-sarcoma**	Cell lines, Tissue & Serum	Tumor *vs*. normal	qPCR	miR-206 (↑)	expression of miR-206 in RMS cells promoted myogenic differentiation and blocked tumor growth	[88]
**Gastrointestinal Stromal Tumor**	Tissue	Tumor *vs*. normal	qPCR	miR-221 (↓) miR-222 (↓)	Regulates cKIT	[89]
**Ewing's Sarcoma**	Cell lines	Primary sarcoma *vs*. Progenitor cells	miRNA Profiling	miR-145 (↓)	miR-145 inhibits stem cell transcription factors Oct4, Sox2, Klf4 and Myc	[90]
**Schwannoma**	Tissue, cell lines	Tumor *vs*. Normal	Microarray, qPCR	miR-7 (↓)	Inhibited expression of Ack1, Pak1, and EGFR	[91]
**MPNST**	Tissue	MPNST *vs*. neurofibroma	Microarray, qPCR	miR-34a (↓)	Partly due to p53 inactivation	[93]
**LEUKEMIA/ LYMPHOMA**						
**Adult T Cell Leukemia**	Cells	primary ATL cells *vs*. normal CD4+ T cells	Microarray	miR-31 (↓)	miR-31 is a suppressor of NIK and pathway involving polycomb-mediated miRNA silencing and NF-kB activation	[94]
**Acute promyelocytic leukemia **	Cells	Leukemia *vs*. Normal Promyelocytes	qPCR	miR-15b (↓) miR-16 (↓) miR-107 (↓) miR-223 (↓) miR-342 (↓) and let-7c (↓)	PML/RARa binds the regulatory sequences of the intragenic miR-342 and let-7c	[95]
**AML **	Cell lines	AML, Human myeloid, CLL cell lines	qPCR microarray	miR-34b (↓)	Cyclic AMP-Responsive Element Binding Protein down-regulation	[96]
**CLL**	Peripheral blood mononuclear cells	Cancer cells *vs*. normal cells	qPCR	miR-92 (↑)	Abnormal elevation of HIF-1α, the key upstream regulator of VEGF	[97]
	Peripheral Blood CD19+ cells	Prognostic factors	qPCR Western blot	miR-29c (↓) miR-223 (↓)	Regulates the Tcl1 oncogene Down-regulation of miR-29 inversely correlates with DNMT expression	[98]
**Hodgkins lymphoma**	Cancer cell lines	Hodgkins *vs*. B cell non Hodgkins	qPCR Microarray	miR-155 (↑)	IKBKE, ZNF537, ZIC3, FGF7, and AGTR1 are functional targets of miR-155	[34]
**Diffuse Large B-cell lymphoma**	Serum	Tumor *vs*. Normal	qPCR	miR-15a (↑) miR-16-1 (↑) miR-29c (↑) miR-155 (↑) miR-34a (↓)	miR-155 directly down regulates one of the MYC antagonists like MAD1, MXI1, ROX/MNT	[99]

Upregulation and downregulation of microRNAs are denoted by (↑) and (↓) arrows respectively.

## 5. miRNA Detection in Body Fluids and Stability

In an attempt to establish miRNAs as biomarkers in cancer, considerable interest has been drawn to the ability of detecting these circulating biomarkers in different body fluids and the stability of miRNAs to resist the numerous extracellular enzymes present in human body. Weber *et al.*, showed the presence of miRNAs in different body fluids such as plasma, saliva, tears, urine, amniotic fluid, colostrum, breast milk, bronchial lavage, cerebrospinal fluid, peritoneal fluid, pleural fluid, seminal fluid and serum [100]. There is also evidence of miRNAs being identified in different body fluids in various cancer types, some of them being serum miRNAs in prostate cancer [101], urine miRNAs in urothelial carcinoma [102], cerebrospinal fluid (CSF) miRNAs in glioma [103], plasma miRNAs in pre-eclampsia [104] and sputum miRNAs in non small cell lung cancer (NSCLC) [105]. These studies provide strong evidence to indicate the existence of miRNAs in different body fluids. The stability of endogenous miRNAs to RNase digestion was shown by adding synthetic miRNAs to the serum from subjects with prostate cancer. The added synthetic miRNAs were destroyed whereas the endogenous miRNAs were able to maintain their stability and were detected in plasma/serum [40]. In yet another study, Chen *et al.* elucidated the stability of miRNAs in various body fluids, after the miRNAs were subjected to a series of experimental conditions such as boiling, low/high pH, extended storage and multiple freeze thaw cycles [57].

Recent findings have demonstrated the presence of miRNAs in exosomes, microvesicles, lipoproteins, apoptotic bodies and large micro particles. Valadi *et al.* analyzed human and mouse mast cell lines and observed the synthesis of new proteins on transfer of exosomal RNA between the donor cells and the recipient cells. They proposed that the exosomes contained both mRNA and miRNA and a possible mechanism of genetic exchange between the cells [106]. It is probable that the stability of endogenous miRNAs is imparted by the protection of miRNAs in membrane bound exosome-like particles called microvesicles [107]. Gallo *et al.* recently observed that a majority of miRNAs in saliva and serum from healthy individuals and systemic lupus erythmatosus (SLE) patients were found to be concentrated in exosomes [108]. 

Evidence from recent studies has also proven the presence of miRNAs in high-density lipoproteins (HDL) [109]. The study by Vickers *et al.* showed that HDL has a major role in endogenous miRNA transport. They isolated HDL from normal human plasma and from patients with familial hypercholesterolemia by density gradient ultracentrifugation and fast-protein liquid chromatography followed by anti-apoA-1 immunoprecipitation. Total RNA was extracted from purified HDL and miRNA profiles analyzed using microarrays. They demonstrated occurrence of various miRNAs in HDL molecules, and one of the abundant miRNA found in both human and mouse HDL was miR-223 [110]. Furthermore, they evaluated dynamic response by subjecting various cell lines to stress. The results revealed a number of proteins involved in the exportation process and suggested nucleophosmin 1 (NPM1) as a protective protein guarding miRNA from degradation. These findings about the stability of endogenous miRNA and protection from degradation outside the cellular environment imparted by exosomes and protective proteins suggest possible reasons for their detection in plasma/serum. 

### 5.1. miRNA Extraction and Quantifying Methods

Extensive studies have implicated miRNAs to be involved in initiation, progression, malignant transformation, prognosis and outcome of cancer. Since the extraction levels of miRNAs directly reflect tumor growth, metastatic potential and therapeutic response, the major challenge lies in determining the presence of miRNAs in different tissues, cell types and body fluids. The result is the development of innovative tools to detect the presence and demonstrate the expression of miRNAs. 

Currently, there are various strategies for miRNA profiling. The widely followed methods for RNA extraction are using the mirVana miRNA extraction kit (Ambion), the Trizol reagent protocol (Invitrogen) and the miRNeasy Mini Kit (Qiagen). Several studies have used the above methods and have introduced few modifications such as double phenol-chloroform extraction in mirVana PARIS protocol [40] and phenol-chloroform extraction methods with or without proteinase K incubation in Trizol reagent protocol [111]. Once isolated as total RNA, miRNAs are further quantified by methods such as *in situ* hybridization, northern blotting, qRT-PCR [112], microarrays, high-throughput sequencing and bead-based arrays [113]. The northern blot analysis and *in situ* hybridization were the earlier methods used for miRNA analysis to elucidate details on miRNA maturation and to highlight the complementarity between a miRNA and its RNA target. However, it is labor-intensive and not amenable for large-scale experiments. The qRT-PCR is based on the quantitative relationship between the amount of starting target present in the assay and the amount of PCR product at any given cycle number. The higher the quantity of initial nucleic acid target present, the earlier the cycle number when the required PCR product amount is achieved. 

A highly specific, sensitive and cost-effective qRT-PCR approach, called the miR-Q, has been developed which does not employ fluorochromic probes or locked nucleic acid (LNA)—modified oligonucleotides. Another convenient method of PCR is the poly (A)-tailed universal reverse transcription. However, all the above methods are low throughput miRNA profiling methods and more recently a high throughput method called Taqman low-density array cards, which allows simultaneous quantification of hundreds of miRNAs using megaplex stem-loop primer pools, have been developed. 

Cummins *et al.* proved the existence of more miRNAs than those that were already known by developing a new approach called miRNA serial analysis of gene expression (miRAGE). The results of the study showed substantial evidence for the presence of miRNAs in colon cancer and many unrecognized forms of known miRNAs [114]. The bead-based arrays are based on impregnating glass beads with different concentrations of fluorescent dye. Different oligonucleotide sequences are attached to each bead, and thousands of beads can be self-assembled on the fiber bundle. Complementary oligonucleotides present in the sample bind to the beads, and bound oligonucleotides are measured by using a fluorescent label. Nelson *et al.* described the RNA-primed array-based Klenow enzyme (RAKE) assay [115]. This method involves the application of klenow fragments of DNA polymerase I to extend unmodified miRNAs hybridized to immobilized DNA probes. It allows rapid expression and simultaneous detection of all known miRNAs in samples. The microarray analysis is the most widely used technique for the genome-wide miRNA profiling and depends on hybridization between the target molecules and the complementary probes. This method is advantageous as it analyzes large numbers of miRNAs and their expression profile at the same time. More recently, nanotechnology based methods have been employed which are based on the electrical detection techniques. With the rapidly emerging role of miRNAs in cancers the expectations to detect miRNAs in tumor tissues with high specificity and sensitivity also keep escalating. Even though, there is no exclusive method available for miRNA extraction, which is rapid, involving easy protocols with minimal requirement of starting material that is also cost effective, the above-mentioned approaches all have great potential in detecting miRNAs in tumor tissues/cells with good amount of sensitivity and specificity.

With regard to miRNA isolation from body fluids, the miRCURY RNA isolation kits from Exiqon may overcome some of the common challenges encountered during isolation of RNA from bio-fluids. Nearly 50 ng RNA can be isolated from plasma separated from 10ml of whole blood sample and addition of carrier RNA such as MS2 phage RNA improves miRNA purification. The kit also suggests the use of RNA spike-in from Exiqon as a method of quality control. Other methods based on mirVana PARIS kit (Ambion) and miRNeasy Mini Kit (Qiagen) are also available, which involves sample disruption followed by an organic extraction process and washing steps to extract total RNA including small RNAs. 

### 5.2. Potential Pitfalls in Developing miRNAs as Circulating Biomarkers

Although recent findings exemplify the major role of miRNAs in cancer diagnosis, their applications in clinical setting is still a work in progress. Even though there is good evidence suggesting the presence of miRNAs in various body fluids during cancer development, the current focus is in developing biomarkers using serum/plasma. However, there are a number of variables to be considered including sample volumes, quality and processing methods. Standardization of sample volume will directly affect the efficacy of miRNA extraction process, which will be the next pre-analytic variable. There are multiple kits available for miRNA extraction, and there has been a contradictions on which reagent gives the maximized miRNA yield. Since serum/plasma fluids are rich in protein there is the possibility of interference with the detection assay. Recent studies suggested the stability of circulating miRNAs from blood ribonucleases as miRNAs being part of exosomes, microvesicles or Ago2 protein [116]. Further analysis is required to see if there are considerable variations in miRNAs released from living cells, which are encapsulated in exosomes/microvesicles as compared to those released from dying or dead cells. Taken together, though there is potential to develop circulating miRNAs as biomarkers, further studies are required to overcome the above-mentioned limitations. 

**Figure 1:**
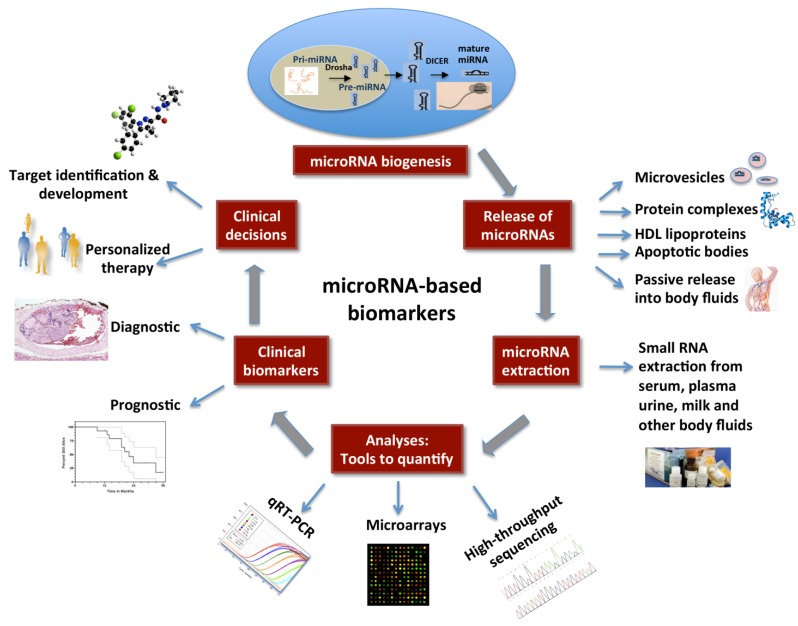
Circulating microRNAs as biomarkers: A schematic diagram showing miRNA biogenesis, modes of their secretion into body fluids, RNA extraction and quantitative approaches. In future, clinical decisions may be made based on the expression levels of miRNAs. This figure is adapted from Steer and Subramanian [117].

The workflow for development of miRNAs as biomarkers is schematically represented in Figure 1. 

## 6. Future Directions

miRNAs are revolutionizing the field of cancer research. These small RNAs are able to distinguish cancer types and subtypes and have the potential to be biomarkers in cancers. However, there is a need to address the level of correlation that exists between the miRNA identified in tumor tissue and circulating miRNAs. Further studies establishing miRNA expression patterns in relation to patient’s age, demography, common health conditions and lifestyles are needed. Before developing serum miRNA as a clinical biomarker, the techniques of miRNA extraction, quantification, data analysis and a normalization strategy needs to be standardized. Currently, one of the main challenges in analyzing miRNA from serum samples is the normalization factor. The effective use of animal models will significantly increase the success of developing suitable miRNA biomarkers in cases where the access to human patient samples is limited [118]. Effectively addressing these challenges will help us overcome some of the current pitfalls and establish miRNAs as fail proof biomarkers in cancer diagnosis and treatment.
